# Harry Francois Devis

**Published:** 1938

**Authors:** 


### HARRY FRANCOIS DEVIS, M.R.C.S., L.R.C.P.

THE death of Dr. H. F. Devis, at the age of 76, removes one of the older students of the Bristol Medical School. A man who in his day played a prominent part in medical as well as municipal politics. He qualified in 1888 after winning at the Bristol General Hospital the Martin Memorial, Clarke and Sanders Scholarships. He was an all-round athlete, being a notable Rugby player and a keen cricketer. He practised for many years in Knowle, and was President of the Knowle Cricket Club. From 1906 to 1913 Dr. Devis represented the Somerset Ward in the Bristol City Council. When Mr. Lloyd George introduced his National Health Insurance Bill in 1911, Dr. Devis was an active participant in the prolonged discussions to which it gave rise between the medical profession and the politicians. Such, however, was his personal charm and forbearing manner that Dr. Devis could always engage in controversy without producing disharmony.

When the National Health Insurance Act became law Dr. Devis retired from practice and became Secretary to the Local Panel Committee and represented this Committee on the Medical Service Committee. In 1926 he retired from Bristol to reside in Marazion, where he died on 8th November last. Dr. Devis leaves a widow and a large family, none of whom, however, chose their father's profession.

